# Characterization and Validation of an “Acute Aerobic Exercise Load” as a Tool to Assess Antioxidative and Anti-inflammatory Nutrition in Healthy Subjects Using a Statistically Integrated Approach in a Comprehensive Clinical Trial

**DOI:** 10.1155/2019/9526725

**Published:** 2019-09-08

**Authors:** Youjin Kim, Sungkyoung Choi, Sungyoung Lee, Saejong Park, Ji Yeon Kim, Taesung Park, Oran Kwon

**Affiliations:** ^1^Department of Nutritional Science and Food Management, Ewha Womans University, Seoul, Republic of Korea; ^2^Department of Applied Mathematics, Hanyang University (ERICA), Ansan 15588, Republic of Korea; ^3^Interdisciplinary Program in Bioinformatics, Seoul National University, Seoul, Republic of Korea; ^4^Department of Sport Science, Korea Institute of Sport Science, Seoul, Republic of Korea; ^5^Department of Food Science and Technology, Seoul National University of Science and Technology, Seoul, Republic of Korea; ^6^Department of Statistics, Seoul National University, Seoul, Republic of Korea

## Abstract

The homeostatic challenge may provide unique opportunities for quantitative assessment of the health-promoting effects of nutritional interventions in healthy individuals. *Objective*. The present study is aimed at characterizing and validating the use of acute aerobic exercise (AAE) on a treadmill at 60% of VO_2max_ for 30 min, in assessing the antioxidative and anti-inflammatory effects of a nutritional intervention. In a controlled, randomized, parallel trial of Korean black raspberry (KBR) (*n* = 24/group), fasting blood and urine samples collected before and following the AAE load at either baseline or 4-week follow-up were analyzed for biochemical markers, ^1^H-NMR metabolomics, and transcriptomics. The AAE was characterized using the placebo data only, and either the placebo or the treatment data were used in the validation. The AAE load generated a total of 50 correlations of 44 selected markers, based on Pearson's correlation coefficient analysis of 105 differential markers. Subsequent mapping of selected markers onto the KEGG pathway dataset showed 127 pathways relevant to the AAE load. Of these, 54 pathways involving 18 key targets were annotated to be related to oxidative stress and inflammation. The biochemical responses were amplified with the AAE load as compared to those with no load, whereas, the metabolomic and transcriptomic responses were downgraded. Furthermore, target-pathway network analysis revealed that the AAE load provided more explanations on how KBR exerted antioxidant effects in healthy subjects (29 pathways involving 12 key targets with AAE vs. 12 pathways involving 2 key targets without AAE). This study provides considerable insight into the molecular changes incurred by AAE and furthers our understanding that AAE-induced homeostatic perturbation could magnify oxidative and inflammatory responses, thereby providing a unique opportunity to test functional foods for antioxidant and anti-inflammatory purposes in clinical settings with healthy subjects.

## 1. Introduction

With people living longer, the attention toward functional foods that promote health maintenance and chronic disease prevention has increased at an alarming rate. Accordingly, to ensure consumer protection from fraud labeling and encourage industry innovation, many governments in global societies have launched strategies to ensure that functional foods promoting health claims in humans are supported by scientific evidence [1]. However, proving health benefits in apparently healthy individuals remains a significant bottleneck that limits the development of functional foods. One of the critical reasons for this difficulty is because the robustness of homeostasis and large interindividuality may easily mask many small and subtle intervention effects [2]. Therefore, a new experimental approach is urgently needed to magnify the effect size of the intervention in a clinical trial.

Temporary and reversible perturbation of homeostasis mimics the early stage of chronic disease, leading to a significant increase in the values of response magnitudes in healthy subjects [3]. The most well-known example is the oral glucose load, which has long been applied to determine the effectiveness of nutritional interventions for improving glucose and insulin metabolism. Recent liquid chromatography-mass spectrometry studies have added other detailed scientific information by characterizing the specific metabolic changes occurring in healthy subjects receiving an oral glucose load [4, 5]. In the meanwhile, over the past 30 years, acute aerobic exercise (AAE) has been applied to test various antioxidant agents with a simple assumption that it may increase the production of reactive oxygen species [6]. However, a comprehensive understanding of how AAE affects physiological processes related to oxidation and inflammation remains unknown [7–11].

This research group recently published data identifying prognostic metabolites for predicting responsiveness to nutritional intervention with Korean black raspberry (KBR) against AAE-induced oxidative stress and inflammation in a placebo-controlled, randomized clinical trial [12]. In the present study, we extend the previous work to characterize and validate the impact of an AAE load of a treadmill exercise at 60% of VO_2max_ for 30 min on the antioxidative and anti-inflammatory effects of a KBR nutritional intervention. First, we conducted a comprehensive investigation into the changes before and following AAE using data obtained from the placebo group. Then, we validated whether the AAE load may be useful to assess the effects of antioxidative and anti-inflammatory nutritional interventions, by comparing the response magnitude to KBR intervention under the conditions with and without the AAE load. In both cases, we determined markers that were differently altered in parallel with the AAE load or KBR intervention, by a linear mixed effect (LME) model analysis of biochemical markers, ^1^H-NMR metabolomics, and RNA-sequencing transcriptomics (RNA-Seq). Finally, we employed the correlation network analysis to obtain system-level insights into the AAE-induced oxidative stress and the effect of KBR on the prevention of oxidative stress and inflammation.

## 2. Materials and Methods

### 2.1. Study Design, Ethical Approval, and Sample Collection

**Figure 1:**
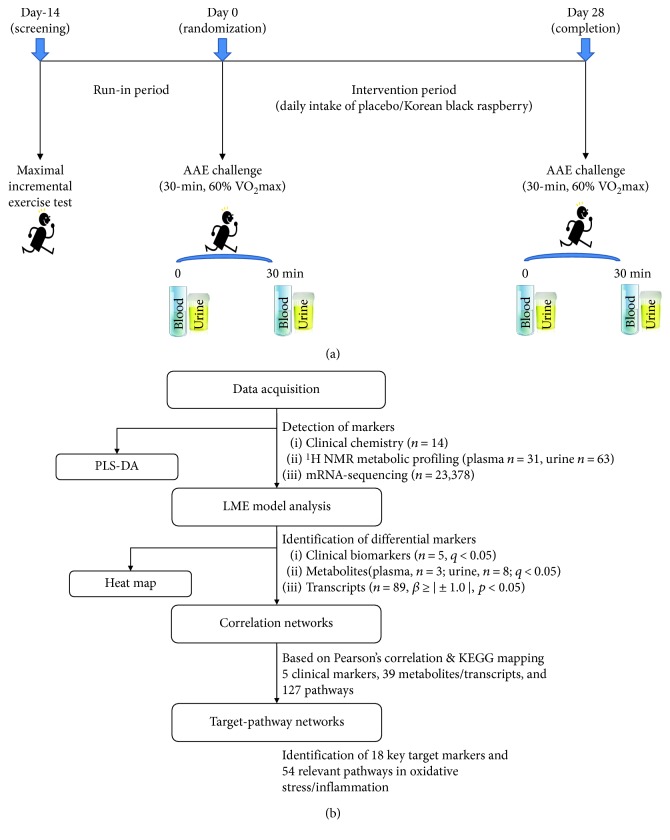
Overview of the study: (a) study design. (b) schematic diagram of statistical analysis.

The experimental protocol, execution, and analytical methods of the intervention study have previously been described in detail [12] and are summarized in Figure 1(a). In short, apparently healthy but overweight (body mass index of 25 ± 2 kg/m^2^) and sedentary (2.5 h/week of exercise) subjects (45 ± 8 years of age) were supplemented freeze-dried powder of KBR or a color/flavor-matched placebo for 4 weeks, in a randomized controlled trial. At baseline and end points, participants fasted overnight before presenting at the laboratory between 08:30 and 10:00 h. After consuming one sachet of placebo or KBR, a single session of AAE was given on a motor-driven treadmill for 30 min at an intensity of 60% of maximal oxygen consumption (VO_2max_). The intensity of exercise was adjusted throughout the protocol by monitoring the participants' heart rate. Blood and urine samples were collected before and immediately after the completion of each exercise session and examined using biochemical analyses, ^1^H-NMR metabolic profiling, and RNA-Seq. The study was approved by the Institutional Review Boards of Ewha Womans University (Seoul, Korea) using procedures in accordance with the revised Helsinki Declaration of 1983. It is registered as KCT0000644 in the WHO International Clinical Trials Registry Platform.

Each participant's VO_2max_ (67 ± 6 mL/kg/min) was determined on the screening day, using a motor-driven treadmill (T150; Cosmed, Rome, Italy) and a computer-controlled respiratory gas analyzer (Quark CPET; Cosmed). The initial velocity was between 4.5 and 6.6 km/h, and the initial grade was between 0.0 and 10.0%. The velocity was increased by 1 km/h, or the grade increased by 2.0% every 2 min until exhaustion. Oxygen uptake was calculated from measures of ventilation, and the oxygen and carbon dioxide in the expired air and the maximal level were determined at or near test completion. Heart rate was measured continuously (Polar T34 transmitter; Polar Electro, Kempele, Finland).

### 2.2. Biochemical Measurements

Plasma malondialdehyde was analyzed by high-performance liquid chromatography. Plasma oxidized low-density lipoprotein, interleukin-6 (IL-6), and tumor necrosis factor-*α* were analyzed by enzyme-linked immunosorbent assays. Activities of lactate dehydrogenase (LDH) and creatine kinase (CK), nitrite/nitrate, and albumin were determined in plasma by spectrophotometric methods. Activities of glutathione peroxidase (GPx), superoxide dismutase, catalase (CAT), and reduced/oxidized glutathione (GSH/GSSG) in erythrocytes were assessed by spectrophotometric methods.

### 2.3. ^1^H-NMR Metabolic Profiling

Plasma and urine samples were sent to the Pusan National University (Busan, Republic of Korea) for nontargeted quantitative metabolome analysis by ^1^H-NMR spectroscopy using a Varian 600 MHz spectrometer (Varian, Palo Alto, CA, USA), as detailed by Kim et al. [12]. The resulting ^1^H-NMR dataset contained 31 metabolites in plasma samples and 63 metabolites in urine samples.

### 2.4. Total RNA Preparation, RNA Sequencing, and Analysis

Whole blood sample was transferred to PAXgene blood RNA tubes (PreAnalytiX, Qiagen/BD, Hilden, Germany) and sent to the Theragen Bio Institute (Suwon, Korea) for total RNA preparation and RNA sequencing on a HiSeq 2500 high-throughput RNA sequencer (Illumina, San Diego, CA, USA). Total RNA was isolated using TRIzol (Invitrogen, Carlsbad, CA, USA). RNA concentrations were quantified using a NanoDrop spectrophotometer (Thermo Scientific, Wilmington, Germany), and the 260/280 nm ratio was confirmed to be between 1.7 and 2.0. The RNA integrity was evaluated using an Agilent 2100 Bioanalyzer system (Agilent, Santa Clara, CA, USA). An RNA integrity number > 7.0 and high-quality RNA (28S/18S > 1) were used for the subsequent experiment. According to the manufacturer's protocol, mRNA molecules were polyA-selected, chemically fragmented, converted into single-stranded cDNA by random-hexamer priming, 3′-end-repaired and adenylated, sequencing adapter-ligated, and polymerase chain reaction-amplified. Libraries for Illumina sequencing were constructed from cDNA. Illumina HiSeq2500 sequencing was conducted according to the manufacturer's specifications, to obtain the 100 bp pair-end sequences. RNA-Seq data were analyzed as described elsewhere [13]. Briefly, reads were mapped against the human reference genome (Ensembl release 72) using STAR 2.3.0e. The transcript expression levels were estimated by the software Cufflinks v.2.1.1, as fragments per kilobase of exon per million fragments mapped, resulting in a set of 23,378 genes. The original gene expression data were log2 normalized.

### 2.5. Statistical Approaches to Data Integration and Pathway Analysis

The statistical analyses were carried out using SAS 9.4 (version 9.4; SAS Institute, Cary, NC, USA) and R (http://www.r-project.org/) with a sequence illustrated in Figure 1(b). Supervised partial least square to latent structure-discriminant analysis (PLS-DA) was used to visualize separation between before and following AAE load conditions for each dataset (biochemical markers, metabolites, and transcripts), using the “ropls” package of the R statistical software version 3.4.2. Explanatory variables were centered at mean before the analysis.

In LME modeling, the repeatedly measured responses, such as “Y” and “*Δ*Y,” were represented as a function of an intercept, where “group” (placebo and KBR; “G”), “AAE load” (before and after AAE; “E”), “period” (week 0 and week 4; “W”), and their interactions. Here, “*Δ*Y” represents absolute changes in markers between baseline and end of the 4-week intervention, and all the covariates were represented as the categorical fixed effect factors. A subject effect (*b*) and random error (*ε*) were included in the following LME model as random variables:

(1) $$\begin{array}{c} {\text{Y}}_{\text{ijkl}}=\beta_{0}+\beta_{1}{\text{G}}_{\text{ij}}+\beta_{2}{\text{E}}_{\text{ik}}+\beta_{3}{\text{W}}_{\text{il}}+\beta_{4}{\text{G}}_{\text{ij}}{\text{E}}_{\text{ik}}+\beta_{5}{\text{E}}_{\text{ik}}{\text{W}}_{\text{il}}+\beta_{6}{\text{G}}_{\text{ij}}{\text{W}}_{\text{il}}+b_{\text{i}}+\varepsilon_{\text{ijkl}},{\text{b}}_{\text{i}}\sim \text{N}\left(0,\sigma_{\text{b}}^{2}\right),\varepsilon_{\text{ijkl}}\sim \text{N}\left(0,\sigma^{2}\right), \end{array}$$

where *Y*_*ijkl*_ is outcome response for subject *i* in group *j* with/without an AAE load *k* at period *l*, and *β*s represent the effects of the covariates on “*E*” (*Y*). A similar model can be derived for “*Δ*Y.” After fitting the LME model, the inference on the main-effect means was made using the R “lsmeans” package [14]. For biochemical markers and metabolites, the *p* values were adjusted for multiple testing by controlling the false discovery rate (FDR); the significance threshold was set to FDR *q* < 0.05. For transcript data, differentially expressed genes were identified if they satisfied both *p* values (<0.05) and were above the absolute value of beta estimate (>0.5). The criterion of beta estimates was equivalent to a fold-change above 1.4-fold and below 0.6-fold. The results were displayed as a heat map using the R “heat map.2” function. For characterization of the AAE load, only the placebo group was included, with adjustment of “period” factor. For validation of the applicability of the AAE load to assess treatment effects, the “period” factor was excluded by using “*Δ*Y”.

Pearson's correlations were calculated from all pairwise comparisons of selected markers and then filtered for correlation coefficient ∣*r*∣ ≥ 0.70. Correlation networks were constructed with Pearson's correlation coefficients as edges, and node size corresponds to the number of edges being aggregated, using the R “igraph” package. We then compared the network topology to identify which were present in one and missing in the other, by assuming that a disparity differentiates characteristics of the state and provides potential clues to the functionality of the process. Next, the target-pathway network was constructed and visualized using Cytoscape (http://www.cytoscape.org). All critical targets in the correlation network were extracted, and their corresponding pathways of oxidative stress and inflammation were retrieved from the Kyoto Encyclopedia of Genes and Genomes (KEGG, http://www.genome.jp/kegg) database. The nodes in the network included key targets and pathways while the edges represented the target-pathway pairs.

## 3. Results

### 3.1. Characterization of the AAE Load

**Figure 2:**
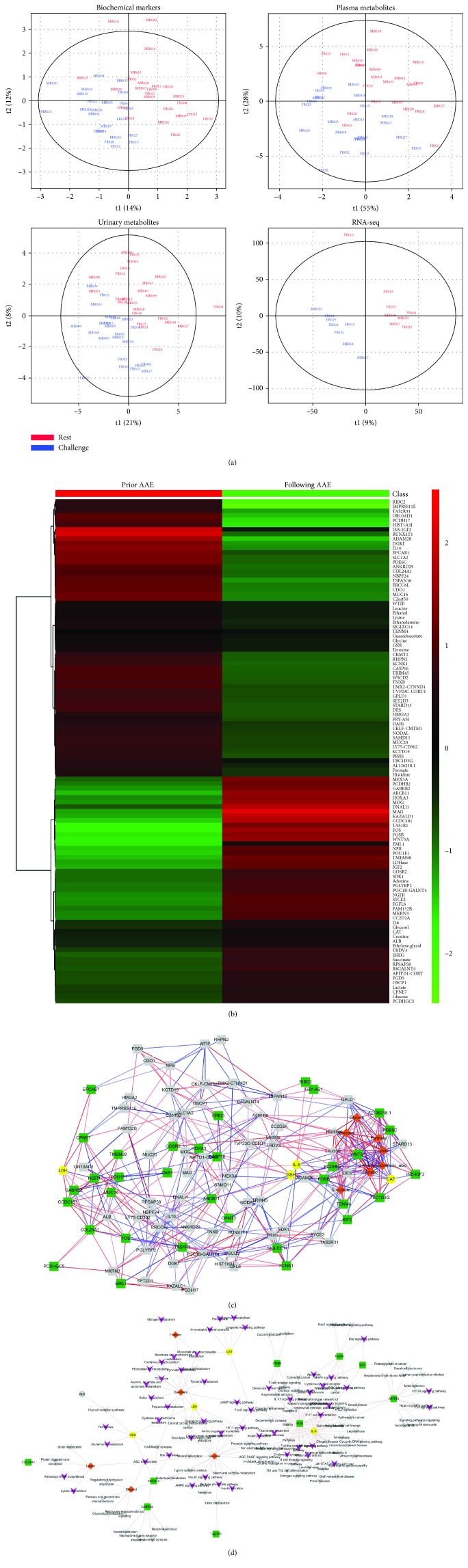
Comprehensive analysis of biochemical and omics data for characterization of AAE load: (a) PLS-DA from biochemical markers, ^1^H-NMR metabolites in plasma and urine, and RNA-sequencing transcripts based on the presence (blue) or absence (red) of AAE load. (b) Heat map of differentially changed biochemical markers, metabolites, and transcripts before and following AAE load. (c) Correlation network between AAE-induced differential markers. The blue and red edges indicate negative and positive correlations. (d) Target-pathway network involved in AAE-induced oxidative stress and inflammation. The nodes in the yellow circle, orange diamond, green square, and purple V-shape indicate biochemical markers, metabolites, transcripts, and pathways, respectively. Grey nodes and lines indicate nonsignificant markers and linkages between targets and pathways, respectively. AAE: acute aerobic exercise; NMR: nuclear magnetic resonance; PLS-DA: partial least squares-discriminant analysis.

The PLS-DA of 14 biochemical, 31 plasma/63 urine metabolomic, and 23,378 transcriptomic data, respectively, demonstrated an apparent separation between before (red) and following (blue) the AAE load in each panel (Figure 2(a)). The following LME model analysis allowed us to identify a total of 105 differential markers that were differently changed by the AAE load, including 5 biochemical markers, 11 metabolites, and 89 transcripts. The information on the estimates, FDR *p* values, and full names is detailed in Supplementary [Supplementary-material supplementary-material-1]. The heat map in Figure 2(b) visualized the overall magnitude of the difference between before and after the AAE load.

Next, Pearson's correlation coefficient was used to test the association among 105 differential markers, generating 50 significant correlations (52% positive and 48% negative) between 5 biochemical markers (yellow), 6 metabolites (orange), and 33 transcripts (green) (Figure 2(c)). The markers with associations were then mapped onto the KEGG pathway database, identifying 127 pathways relevant to the AAE load. Of these, 54 major pathways were annotated to be related to the oxidative stress and inflammation pathways (appear as purple V-shape against grey arrow). Eighteen markers involved in these multiple KEGG pathways were as follows: CAT, GSH, IL-6, and LDH (yellow); formate, glucose, glycerol, lysine, and succinate (orange); and bile salt export pump (BSEP/ABCB11), creatine kinase mitochondrial 2 (CKMT2), epiregulin (EREG), FBJ murine osteosarcoma viral oncogene homolog (FOS), FBJ murine osteosarcoma viral oncogene homolog B (FOSB), insulin-like growth factor 2 (IGF2), nerve growth factor receptor (NGFR), phosphodiesterase 6C (PDE6C), and WNT5A (green) (Figure 2(d) and Supplementary [Supplementary-material supplementary-material-1]).

### 3.2. Validation of the Use of an AAE Load to Magnify the Responses to Nutritional Intervention

**Figure 3:**
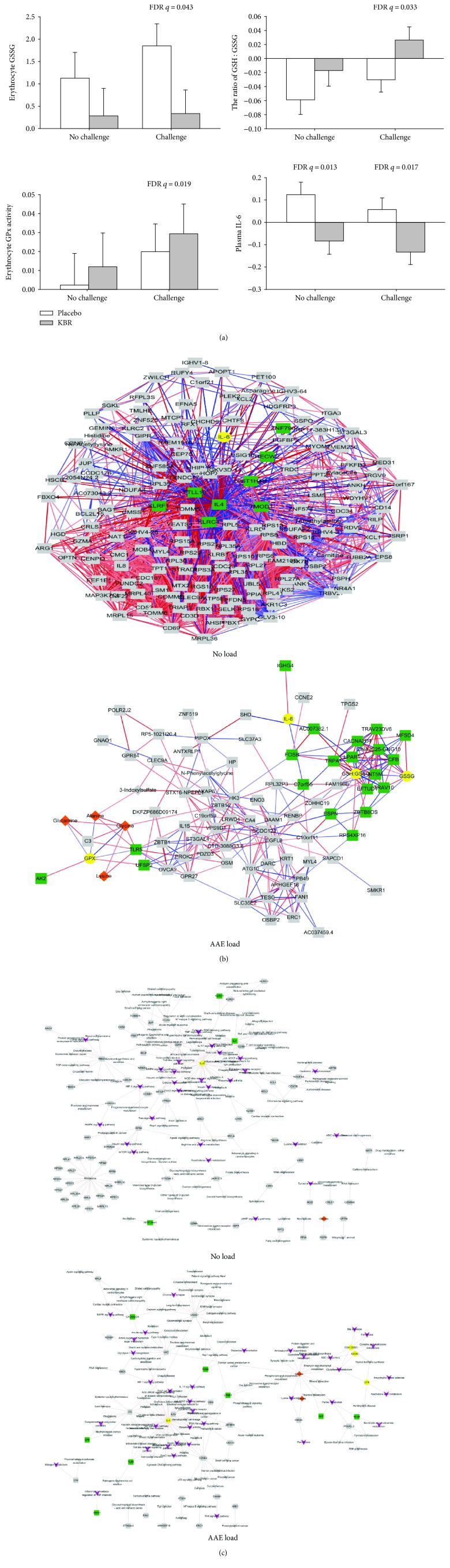
Validation of the use of AAE load using the results of 4-week Korean black raspberry intervention in sedentary overweight subjects by comparing with no load: the AAE load (a) increased the magnification of biochemical effects and decreased the degree of variations in (b) the correlation network and (c) the target-pathway network. The nodes in the yellow circle, orange diamond, green square, and purple V-shape indicate biochemical markers, metabolites, transcripts, and pathways, respectively. Grey nodes and lines indicate nonsignificant markers and linkages between targets and pathways, respectively. AAE: acute aerobic exercise.

The magnitude of biochemical responses to KBR consumption was compared under two conditions (with and without the AAE load), showing that the AAE load provided more quantitative information on the antioxidative biochemical effects of KBR intervention than no load (Figure 3(a)). Antioxidant effects of KBR were significant only under AAE load conditions, as measured by GSSG (*q* = 0.043), GSH : GSSG ratio (*q* = 0.033), and GPx (*q* = 0.019) in erythrocytes, but the anti-inflammatory effect of KBR was similarly significant in either condition, as measured by IL-6 (*q* = 0.013 with no load versus *q* = 0.017 with AAE load). To understand the underlying physiological processes, we determined alterations in metabolomics and transcriptomics under each state using the LME model analysis (Supplementary [Supplementary-material supplementary-material-1]). As a result, we identified 87 differential markers (6 urinary metabolites and 81 transcripts) under the AAE load versus 162 differential markers (7 urinary metabolites and 155 transcripts) under no load condition.

Pearson's correlation coefficient analysis was then performed to identify the associations among all differential markers under each condition. Eight significant correlations (37.5% positive and 62.5% negative) were generated between IL-6 (yellow) and 8 transcripts (green) under no load while 31 significant correlations (51.6% positive and 48.4% negative) were generated between 4 biochemical markers (yellow), 4 metabolites (orange), and 20 transcripts (green) under the AAE load (Figure 3(b)). The full information is detailed in Supplementary [Supplementary-material supplementary-material-1]. Subsequent mapping of the associated markers onto the KEGG pathway database in each condition demonstrated 120 pathways under no load versus 128 pathways under the AAE load. Of these, pathways annotated to be related to oxidative stress and inflammation (appear as purple V-shape against grey arrow) were 12 pathways involving IL-6 (yellow), HIST1H4H, IL-4, and killer cell lectin-like receptor subfamily C1 (KLRC1) (green) under no load versus 29 pathways involving GSSG, GSSG/GSH ratio, GPx, IL-6 (yellow), glycine (orange), adenylate kinase 2 (AK2), calcium channel subunit (CACNA2DA), complement factor B (CFB), lysophosphatidic acid receptor 1 (LPAR1), deoxyribonucleotidase-2 (dNT-2/NT5M), toll-like receptor 5 (TLR5), and transient receptor potential A1 (TRPA1) (green) under the AAE load (Figure 3(c) and Supplementary [Supplementary-material supplementary-material-1]).

## 4. Discussion

To the best of our knowledge, we are the first to characterize and validate the use of an AAE load as a tool to induce oxidative and inflammatory stress, by applying advanced statistical and systems biology techniques to the data obtained from a comprehensive clinical trial of KBR. A PubMed search of the clinical studies published in the last 10 years revealed that the AAE load was used to test the impacts on vascular endothelial function, hemostasis, and cognitive performance, with no knowledge of protocol characteristics. Most studies have been conducted using either treadmill or cycle ergometer exercise at 55–70% VO_2max_ for less than 1 h. In the present study, we utilized a medium duration (30 min) of moderate intensity (60% of VO_2max_) treadmill exercise for the AAE load, expecting to induce oxidative and inflammatory stress without eliciting detrimental oxidative injury such as structural damage of muscle tissue due to physical overexertion [15]. To monitor tissue damage and physiological cell turnover, we determined the release of intracellular enzymes CK and LDH into plasma and found a significant increase of LDH but no changes in CK levels with the AAE load. The CK is an enzyme specific to the muscle and responsible for catalyzing ATP-dependent phosphorylation of creatine, while the LDH is an ubiquitously expressed enzyme responsible for catalyzing anaerobic conversion of pyruvate to lactate [16]. Therefore, the elevated plasma level of CK is used as an early and indirect marker of exercise-induced damage in muscle tissue [17, 18], whereas that of LDH can indicate ubiquitous tissue damage derived from oxidative stress [19].

To assist in understanding the physiological process and quantifying the changes occurring throughout the body in response to KBR consumption with the AAE load in a clinical trial [20], we applied combined transcriptomic and metabolomic analysis. The PBMC gene expression pattern was postulated to be highly correlated with those in various tissues [21, 22] since alterations occurring in individuals throughout the body can leave genetic footprints of physiological changes induced by environmental stresses or dietary interventions, in the blood [23]. Metabolic profiling was also expected to provide further potential insights into the conclusive information regarding the alterations in transcriptomes [24]. Together with omics technologies, advanced statistical analysis considerably strengthened our research. The LME model analysis was used to identify differential biochemical and omics markers. The LME model allows a systematic approach to predict essential outcomes by dealing with the categorical grouping factor and between-subject baseline differences [25, 26] and, thus, has recently captured great attention in clinical trials [27]. Finally, in the hope of gaining insight into the influence of the AAE load and KBR consumption on the interactions of targets from our data and the molecular network, a correlation network analysis was carried out [28]. As a result, we identified various AAE-induced differential molecular markers, including CAT, GSH, IL-6, LDH, formate, glucose, glycerol, lysine, succinate, ABCB11, CKMT2, EREG, FOS, FOSB, IGF2, NGFR, PDE6C, and WNT5A, which were implicated in 54 pathways related to oxidative and inflammatory stress. The selected biochemical markers were consistent with those from previous studies using a similar intensity as our protocol [6, 29]. Of all 54 metabolism and signaling pathways identified in this study, the ABC transporters and bile salt secretion [30, 31], glyoxylate and dicarboxylate [32], pyruvate metabolism [33, 34], FOXO [35], glucagon [32], HIF-1 [36], IL-17 [37], MAPK, and PI3K-Akt [38] signaling pathways were most highlighted by the data mining of published data of the low level oxidative stress.

A complex biological process involved in health and disease can be classified into three stages: the normal, the predisease, and the disease. Chen et al. [39] suggested that the predisease stage features larger fluctuations and low resilience and robustness to perturbations as compared with the normal stage. This concept agrees with those of van Ommen et al. [2, 20] and Elliott et al. [40], who proposed that acute challenges might be useful to amplify the degree of homeostatic perturbation and thereby enable quantifying the health status capture. Based on these concepts, we assumed that the AAE load might induce a temporary transition from the normal to the predisease stage, facilitating the quantification of the intervention effect, due to the weakening of the homeostatic robustness. Our assumption was supported by the findings that the AAE load decreased the degree of variations in gene expressions and metabolic regulatory processes, as well as increased the magnification of biochemical effects related to oxidative stress and inflammation as compared with no load. These findings concurred with a recent study by Fazelzadeh et al. [41], who introduced the use of a mixed-meal challenge to better explore the capacity of coping with metabolic stressors.

Furthermore, in this study, we proved that AAE-induced oxidative stress, as evidenced by the changes in GSH redox status, was reversed by KBR intervention, suggesting that the AAE load appropriately targets oxidation and inflammation. The redox balance is commonly assessed via the dynamic interplay of the GSH-GSSG couple in conjunction with GSH-dependent enzymes [6, 36]. The GSH, a major intracellular nonprotein sulfhydryl (-SH) compound, plays an important role as the first line of direct defense against oxidative stress [42]. The reactive oxygen species generated under oxidative stress conditions might cause GSH oxidation, leading to GSSG generation and the intracellular decrease in the levels of GSH and its dependent enzymes [43]. In this way, erythrocyte GSH protects hemoglobin and other cellular constituents from oxidation that causes changes in the micromembrane elasticity or whole cell deformability [44]. The changes in GSH redox status were further supported in this study by the alterations of glutamate-centered metabolism, including the alanine, aspartate,and glutamate metabolism, and the lysine degradation, which, together, were considered to be the most relevant pathways involved in oxidative stress [45, 46] and inflammation [47].

In the meantime, we found that the KBR intervention significantly reversed the AAE load-induced oxidation of GSH and its related enzymes. This result was further explored by the integrated pathway network analysis, to provide insight into the molecular changes. The GSH metabolism [48, 49], ABC transporters [30, 50], HIF-1 [36], and Jak-STAT [51] signaling pathways were found to be involved in the restoration of erythrocyte GSH balance and relevant oxidative stress. Taken together, based on the previously established concepts [20], we validated that the AAE load can serve a valuable tool to advance our knowledge in developing nutritional interventions against oxidative and inflammatory stress for maintaining health and preventing disease. For example, now we could extend our study to claim that the KBR intervention may help to improve oxidative stress by restoring the erythrocyte GSH balance.

It is notable that the limitation of the present study was not to measure the exogenous metabolite (food metabolome) alterations in biosamples obtained by the proposed method. Walton et al. [52] reported that antioxidant capacity might be attributed to the metabolites of phytochemicals rather than the phytochemicals themselves. Simultaneous exogenous and endogenous metabolite profiling will be exceptionally informative when coupled with a nutrition system approach and can be used to provide explanations for the synergistic mechanisms of action. Furthermore, considering the catalytic cycle of redox reaction involves several enzymes, such as xanthine oxidase, GSH reductase, and GPx [36]; analysis of single nucleotide polymorphisms for mutations in these enzymes would be helpful to identify subjects with the highest likelihood of benefit from an intervention.

## 5. Conclusions

The attention toward functional foods has brought challenges for the development of a new experimental methodology in a human clinical trial. This study sought to define the AAE load (treadmill exercise at 60% of VO_2max_ for 30 min) that could reliably cause a temporary and reversible perturbation of homeostasis by using omics platforms (metabolomics and transcriptomics) and statistical modeling. Findings from the present study suggest that the AAE load can be utilized as a homeostatic load model, reaffirming the previous findings and providing physiological and molecular mechanisms involved in the regulation of oxidative and inflammatory stress. It was also subsequently proved that the AAE load could be exploited to detect the beneficial effect of a nutritional intervention against oxidative and inflammatory stress in apparently healthy subjects. This AAE load may have further implications for defining appropriate surrogate markers of disease progression related to oxidative and inflammatory stress, validating experimental nutritional interventions and discovering prognostic molecular biomarkers for predicting the response to nutritional intervention. Further expansion of the exercise challenge model could also be performed to drive the development of new nutritional interventions related to the other health promotion (for example, a resistance exercise model for muscle protein synthesis).

## Data Availability

The data used to support the findings of this study are available from the corresponding authors upon request.

## Supplementary Materials

Supplementary Materials Supplementary Table S1: list of differential markers before and following AAE load. Supplementary Table S2: KEGG pathways related to oxidative and inflammatory stresses identified by AAE load. Supplementary Table S3: list of the differentially expressed genes and metabolites that best describe transcriptomic changes after KBR intervention with or without AAE load. Supplementary Table S4: correlations (≥0.7 or ≤-0.7) among differential markers after KBR intervention under no and AAE load. Supplementary Table S5: KEGG pathways related to oxidative stress after KBR intervention with and without AAE load.
